# The Predictive Value of Glasgow-Blatchford Score: The Experience of an Emergency Department

**DOI:** 10.7759/cureus.34205

**Published:** 2023-01-25

**Authors:** Penélope Correia, Ana Spínola, Joana F Correia, Ana Marta Pereira, Mário Nora

**Affiliations:** 1 General Surgery, Centro Hospitalar de Entre Douro e Vouga, Santa Maria da Feira, PRT; 2 Immunohemotherapy, Centro Hospitalar de Entre Douro e Vouga, Santa Maria da Feira, PRT; 3 General Surgery, Unidade Local de Saúde de Matosinhos, Matosinhos, PRT

**Keywords:** outpatient, cut-off value, glasgow-blatchford score, emergency department, gastrointestinal bleeding

## Abstract

Introduction: Upper gastrointestinal bleeding (UGB) is a common emergency and a major cause of morbidity and mortality worldwide. An early and accurate assessment at admission is essential to estimate the severity of each case, assisting in the management of patients. The Glasgow-Blatchford score (GBS) is currently recommended for risk stratification of UGB in the emergency department (ED), helping triage patients to in-hospital vs. ambulatory management. The aim of this study was to test the validity of the GBS in an ED.

Methods: Patients who presented to the ED with a diagnosis of UGB between 2017 and 2018 were retrospectively analyzed.

Results: The mean GBS value of the 149 patients included in the study was 10.3. Of the patients, 4.3% had values ≤1 and 8.7% had values ≤3. The sensitivity and negative predictive value for intervention needs (98.9% and 91.7%) and complications in 30 days (100% and 100%) remained high with a threshold ≤3. In the receiver operating characteristic curves, GBS presented an area under the curve of 0.883 and 0.625, regarding the need for intervention and complications in 30 days, respectively.

Conclusions: In our population, the threshold ≤2, and eventually ≤3, allows the identification of twice as many low-risk patients, manageable as outpatients, without significant increases in intervention needs or complications in 30 days.

## Introduction

Upper gastrointestinal bleeding (UGB) is a common emergency with an incidence of 100 cases per 100,000 adults per year and is a major cause of morbidity and mortality worldwide, the latter reaching 2-10% [1,2]. It is defined as an intraluminal hemorrhage proximal to the ligament of Treitz, whose clinical presentation can vary from a slight, self-limited hemorrhage (up to 60% of cases), to a massive, life-threatening hemorrhage [3]. Thus, an early and accurate assessment at admission is essential to estimate the severity of each case, assisting in the clinical guidance of patients (outpatient or inpatient surveillance, level of in-hospital care required, and endoscopy timing).

Several scoring systems have been developed for the assessment of patients with UGB to predict clinical outcomes. The most frequently used are the Rockall score, the Glasgow-Blatchford score (GBS), and AIM65. However, more recently, and after numerous comparative studies, international guidelines consider that GBS more accurately predicts the need for intervention (transfusion, endoscopic, or surgical treatment), re-bleeding, and mortality, due to its high sensitivity (approximately 99%) in identifying high-risk patients [4-6].

Designed and published in 2000 by Blatchford et al., the GBS is based on clinical and laboratory variables, enabling its use prior to endoscopy, making it useful and easily applicable in the emergency department (ED), including in less differentiated hospitals [7]. According to the referred guidelines, patients with GBS ≤ 1 can be monitored on an outpatient basis since they will rarely need any type of intervention, corresponding to about 20% of patients with UGB admitted at the ED [2]. Lately, some studies have questioned and proved the safety of broader thresholds, allowing the identification of a greater number of low-risk patients, who may be safely discharged from the ED, namely, those with GBS ≤ 2, recognizing that there is still some controversy over the most appropriate threshold [3,8,9].

The aim of this study is to verify the validity of the GBS in the orientation of patients with UGB evaluated in an ED.

## Materials and methods

This is a retrospective study carried out at a secondary care hospital center responsible for providing healthcare services to approximately 340,000 habitants.

The International Classification of Diseases, Ninth Revision (ICD-9) codification was used to identify patients with UGB admitted in the ED between January 2017 and December 2018: gastrointestinal hemorrhages (578) (hematemesis (578.0), blood in the stool (578.1), and unspecified (578.9)), malignant neoplasm of the stomach (151), malignant neoplasm of the colon (153), malignant neoplasm of rectum rectosigmoid junction and anus (154), gastric ulcer (531), duodenal ulcer (532), peptic ulcer, site unspecified (533), gastritis and duodenitis (535), bleeding rectal (569.3), angiodysplasia intestine/stomach and duodenum with hemorrhage (569.85/537.83), and Dieulafoy lesion (569.86/537.84). Inclusion criteria for this study were age over 18 years old and clinical presentation compatible with UGB, such as hematemesis, melena, or hematochezia. Patients with a UGB diagnosis in the previous 30 days were excluded.

Demographic and clinical data, as well as information regarding diagnostic and therapeutic procedures, and clinical orientation (discharge vs. hospital admission) at ED, were collected through the patient’s electronic clinical file.

Since our hospital does not have gastroenterology services available 24 hours a day, some patients were transferred to other hospitals, and some of them eventually stayed there. In these cases, the information regarding diagnostic examinations and treatments performed was retrieved through an electronic national health registry available online. The same platforms were utilized to ascertain the admission for rebleeding and mortality in the following 30 days. Only the mortality directly related to an episode of digestive hemorrhage was considered.

In the descriptive analysis of demographic and clinical data, the categorical variables are presented in the form of frequencies and percentages, and continuous variables as averages with the respective standard deviations. Estimates of sensitivity, specificity, positive predictive value (PPV), and negative predictive value (NPV) were calculated with a 95% confidence interval (CI). The prognostic accuracy of GBS concerning the endpoints of need for intervention (transfusion, endoscopic, and/or surgery hemostasis) and readmission for rebleeding and/or mortality at 30 days was evaluated by creating receiver operating characteristic (ROC) curves and calculating the area under the curve (AUC). Statistics analysis was performed using the Statistical Package for the Social Sciences (SPSS) version 25.0 (IBM Corp., Armonk, NY).

## Results

Of the 463 patients admitted with the ICD-9 code of UGB, only 158 had a compatible clinical presentation. Nine patients were excluded from the study for having the same diagnosis at the ED in the previous 30 days. A total of 149 patients were included in the study. Demographic, clinical, and analytical data are represented in Table 1.

**Table 1:** Demographic, clinical, and analytical data

Demographic, clinical, and analytical data	
Age, years	69 ± 16
Men, % (n)	62.4% (93)
Melena, % (n)	55% (82)
Hematemesis, % (n)	32.2% (48)
Melena + hematemesis, % (n)	10.7% (16)
Hematemesis + hematochezia, % (n)	2% (3)
Syncope, % (n)	9.4% (14)
Comorbidities	
Chronic liver disease, % (n)	15.4% (23)
Heart failure, % (n)	20.1% (30)
Medications	
Antiplatelet therapy, % (n)	28.2% (42)
Oral anticoagulation, % (n)	18.8% (28)
Hemoglobin at admission, g/dL	9.3 ± 2.9
Glasgow-Blatchford score (n = 138)	10.3 ± 4.4

At the ED admission, the most frequent presentation was melena (55%). Syncope associated with blood loss was present in 9.4% (n = 14) of patients. According to medical records, 15.4% (n = 23) of patients suffered from cardiac failure and 20.1% (n = 30) from chronic liver disease. The mean hemoglobin level was 9.3 ± 2.9 g/dL (3.4-16.5). According to Blatchford et al.'s criteria [7], the average of GBS was 10.3 ± 4.4 (0-19); 4.3% (n = 6) had values ≤ 1, and 8% (n = 11) had values ≤ 2. Calculation of GBS was not possible in 11 patients for insufficient data (Table 1).

Red blood cell transfusion was performed in 59.1% of patients (n = 88), with a mean of 2.5 ± 1.2 units (one to seven) transfused during their stay in the ED.

Diagnostic upper gastrointestinal endoscopy was performed in 77.7% of the patients (n = 115), and hemostatic techniques were needed in 34.8% (n = 40) of cases (Table 2). The most frequently identified diagnoses were gastric (18.3%, n = 21) and duodenal (15.7%, n = 18) ulcers and esophageal varices (10.4%, n = 12) (Table 3). Endoscopic hemostatic techniques were not successful in two cases, which were surgically addressed.

**Table 2:** Orientation

Orientation (n = 149)	
Endoscopy, % (n)	77.7% (115)
Intervention, % (n)	64.4% (96)
RBC transfusion, % (n)	59.1% (88)
Units	2.5 ± 1.2
Endoscopic hemostasis, % (n)	34.8% (40)
Surgery, % (n)	1.3% (2)
Medical discharge, % (n)	35.6% (53)
Hospital stay, % (n)	61.7% (92)
Duration, days	8 ± 6
30 days complications	15.4% (23)
Readmission, % (n)	9.4% (14)
Mortality, % (n)	
Total	6% (9)
Upper gastrointestinal bleeding related	2% (3)

**Table 3:** Endoscopic diagnosis

Endoscopic diagnosis (n = 115)	
Gastric ulcer, % (n)	18.3% (21)
Duodenal ulcer, % (n)	15.7% (18)
Esophageal varices, % (n)	10.4% (12)
Erosive gastritis, % (n)	7.8% (9)
Hypertensive gastropathy, % (n)	4.3% (5)
Gastric neoplasm, % (n)	4.3% (5)
Mallory-Weiss syndrome, % (n)	3.5% (4)
Other, % (n)	15.7% (18)
Without hemorrhagic focus, % (n)	20% (23)

Of the 149 patients evaluated in the ED, 35.6% (n = 53) were discharged and 61.7% (n = 92) were hospitalized, with a mean hospital stay of 8 ± 6 days (Table 2).

Thirty days after the first hemorrhagic event, 9.4% (n = 14) of patients returned to the ED due to hemorrhagic recurrence, which had a mean GBS value in the initial episode of 11.1 ± 3.2 (6-15). Nine patients (6%) died, three (2%) of them from a cause directly related to UGB (Table 2), showing values of 13, 14, and 17 in the GBS at the time of the initial hemorrhagic episode.

**Figure 1:**
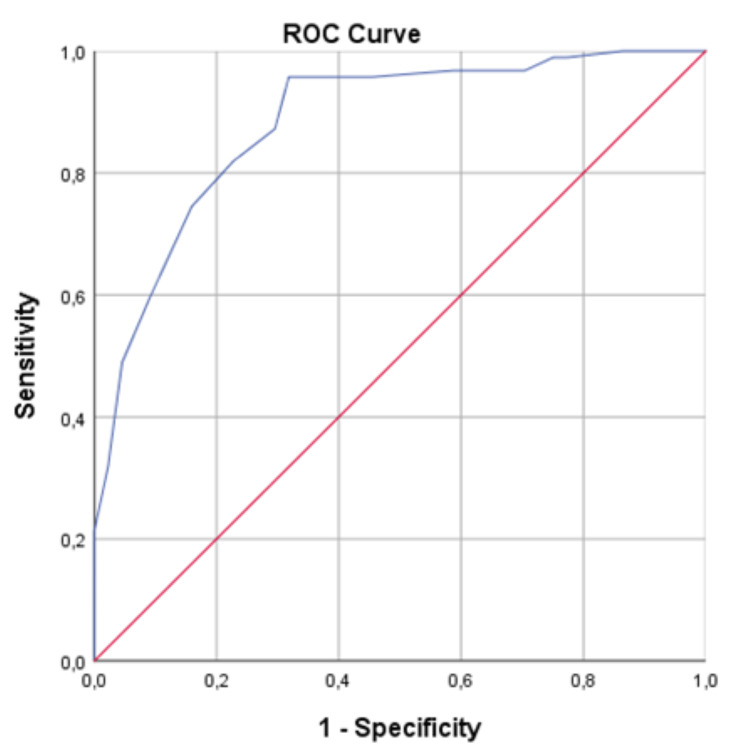
ROC curve concerning the need for medical-surgical intervention (transfusion/endoscopic hemostasis/surgery) ROC: receiver operating characteristic.

**Figure 2:**
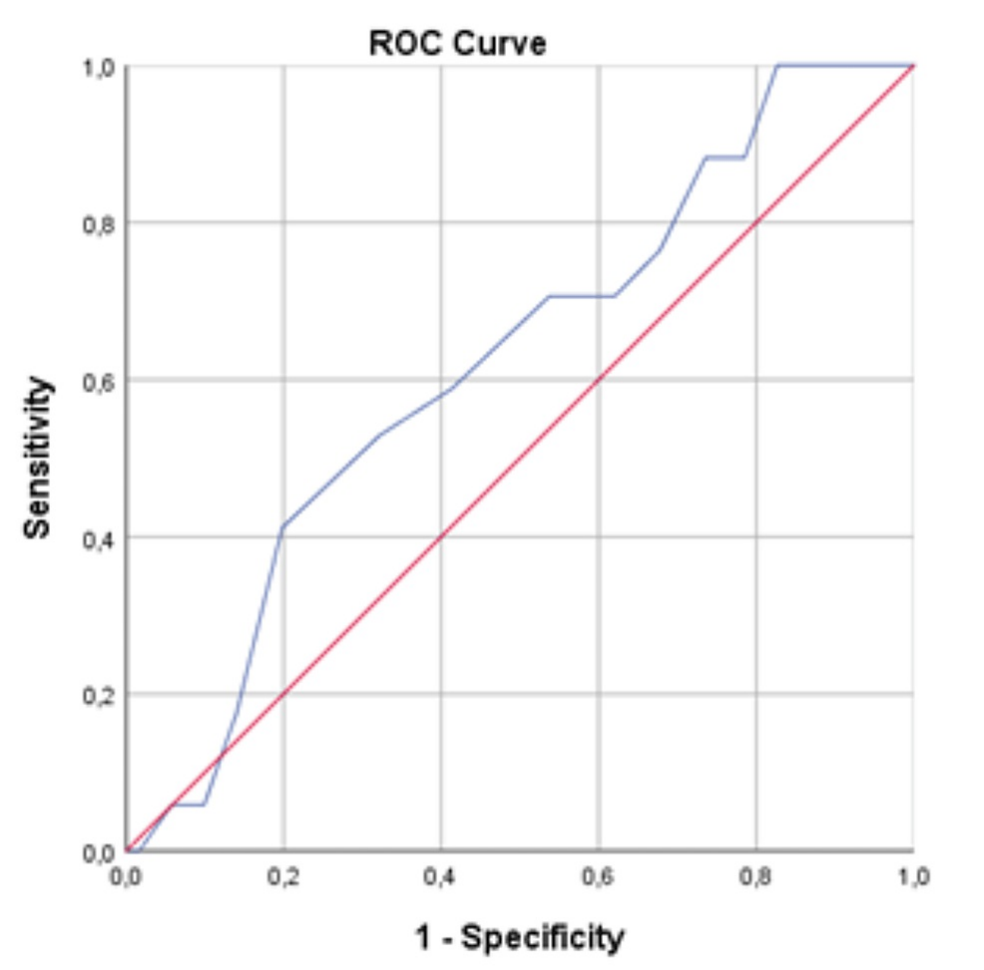
ROC curve concerning complications in 30 days (readmission and mortality) ROC: receiver operating characteristic.

From the analysis of the ROC curves, the GBS proved to be a good discriminator of the need for intervention (Figure 1), with an AUC of 0.883 (95% CI: 0.823-0.943). The same discriminatory power was not verified in relation to the predictability of readmission due to hemorrhagic recurrence and mortality in 30 days (Figure 2), whose AUC was 0.625 (95% CI: 0.501-0.750). Tables 4, 5 show the sensitivity, specificity, PPV, and NPV associated with increasing values in the GBS for the need for intervention (transfusion, endoscopic hemostasis, and/or surgery) and for hemorrhagic recurrence and mortality associated with the UGB in the 30 days after the initial hemorrhagic event, respectively.

**Table 4:** Number of patients who needed medical-surgical intervention (n = 138) GBS: Glasgow-Blatchford score; PPV: positive predictive value; NPV: negative predictive value.

GBS	Patients	Interventional	Sensitivity	Specificity	PPV	NPV
0	4 (2.9%)	0	100%	9.1%	70.1%	100%
≤1	6 (4.3%)	0	100%	13.6%	71.2%	100%
≤2	11 (8%)	1	98.9%	22.7%	73.2%	90.9%
≤3	12 (8.7%)	1	98.9%	25%	73.8%	91.7%
≤4	16 (11.6%)	3	96.8%	29.5%	74.6%	81.3%
≤5	21 (15.2%)	3	96.8%	40.9%	77.8%	85.7%
≤6	28 (20.3%)	4	95.7%	54.5%	81.8%	85.7%
≤7	34 (24.6%)	4	95.7%	68.2%	86.5%	88.2%
≤8	43 (31.2%)	12	87.2%	70.5%	86.3%	72.1%

**Table 5:** Number of patients with readmissions or mortality in 30 days (n = 138) GBS: Glasgow-Blatchford score; PPV: positive predictive value; NPV: negative predictive value.

GBS	Patients	Readmission/mortality	Sensitivity	Specificity	PPV	NPV
0	4 (2.9%)	0	100%	3.3%	12.7%	100%
≤1	6 (4.3%)	0	100%	5%	12.9%	100%
≤2	11 (8%)	0	100%	9.1%	13.4%	100%
≤3	12 (8.7%)	0	100%	9.9%	13.5%	100%
≤4	16 (11.6%)	0	100%	13.2%	13.9%	100%
≤5	21 (15.2%)	0	100%	17.4%	14.5%	100%
≤6	28 (20.3%)	2	88.2%	21.5%	13.6%	92.9%
≤7	34 (24.6%)	2	88.2%	26.4%	14.4%	94.1%
≤8	43 (31.2%)	4	76.5%	32.2%	13.7%	90.7%

## Discussion

UGB is a common cause of ED admission, with a broad spectrum of severity. Despite improvement in the therapeutic options, high morbidity and mortality are still observed in these patients.

Thus, an accurate assessment at admission is essential to predict the severity of each case, allowing the identification of patients who will need hospitalization or invasive procedures, as well as low-risk patients who can be safely discharged, reducing unnecessary hospitalizations. This early stratification is particularly useful in institutions with no gastroenterology available 24 hours a day, to sort patients who require prompt transfer to other institutions or wait for endoscopic evaluation.

The risk scales applied in these contexts must have high sensitivity and specificity. To achieve higher levels of safety, the value of sensitivity is crucial as it prevents high-risk bleeding patients from being misinterpreted as low-risk when considering early discharges. Specificity is not as important since low values translate into unnecessary hospitalizations for low-risk patients and, therefore, without deleterious repercussions on patients' safety.

Regarding demographic and diagnosis characterization, our population shows to be similar to those described in other studies, with a higher percentage of male patients (62%), a mean age of 69 years old, and gastric and duodenal ulcers being the most frequent endoscopic diagnosis [10,11].

One of the findings that stand out in our study is the reduced number of patients admitted to the ED with GBS ≤ 1, corresponding to only 4.3%, contrasting to around 20% reported in the literature [2]. This fact can be explained by the study design and population's characteristics as well as the geographic area covered by the hospital center. First, not all patients were correctly identified according to the ICD-9 codification, more often failing in mild cases. Second, once the geographic area includes distant and rural places, many of the low-risk patients seek medical care in small private clinics or general physicians, therefore not being referred to the hospital center. This limitation was also noted in other studies, such as Laursen et al. (2015) [12]. Nevertheless, the study corroborates that the GBS with a threshold ≤ 1 is in fact safe in the identification of low-risk patients.

Regarding the need for medico-surgical interventions, the AUC was 0.883 (95% CI: 0.823-0.943), reflecting a good discriminative power, in line with the literature, with a sensitivity and NPV of 100% to values ≤ 1. This ability was not so obvious in the prediction of complications in 30 days, with an AUC of 0.625 (95% CI: 0.501-0.750); however, for values ≤ 1, there were no readmissions due to hemorrhagic recurrence or mortality reported [13,14].

To increase the proportion of low-risk patients, without compromising the sensitivity and safety of the scale, several studies have proposed the use of a cut-off ≤ 2 in the GBS [3,8,9,15,16]. According to our results, for a threshold ≤2, and eventually ≤3, the sensitivity and NPV remain high (98.9% and 90.9% for ≤2 and 98.9% and 91.7% for ≤3, respectively). There was only one case (0.7%) of a 33-year-old man presenting with hematemesis secondary to a Mallory-Weiss syndrome without hemodynamic or analytical repercussions, who needed hemostatic endoscopic intervention. No cases of recurrence or mortality in 30 days were described (sensitivity and NPV of 100%).

There are limitations in this study that need to be acknowledged, namely, the retrospective nature and the small sample size. Also, as previously mentioned, the hospital location may also have biased the results, possibly contributing to a decreased percentage of mild UGB with potential for outpatient guidance, though it demonstrates more real applicability of the GBS, adjusted to the most frequent reality of hospital centers in our country.

## Conclusions

GBS is a simple and reliable tool for application in the ED to discriminate between high-risk UGB patients who require hospitalization and/or endoscopic intervention from low-risk patients who can be safely discharged, potentially reducing costs associated with unnecessary interventions and hospitalizations, without compromising patients' safety.

This study corroborates what has been hypothesized in other publications that more comprehensive thresholds of the GBS allow the identification of a greater number of patients who can be safely discharged, without significant increases in the need for interventions or complications. The applicability of slightly higher thresholds seems to be even more useful in hospitals without a gastroenterologist available 24 hours.
